# Possible Mechanisms Underlying the Therapeutic Effects of Transcranial Magnetic Stimulation

**DOI:** 10.3389/fnhum.2015.00303

**Published:** 2015-06-16

**Authors:** Alexander V. Chervyakov, Andrey Yu. Chernyavsky, Dmitry O. Sinitsyn, Michael A. Piradov

**Affiliations:** ^1^Research Center of Neurology, Moscow, Russia; ^2^Moscow Institute of Physics and Technology, Russian Academy of Sciences, Moscow, Russia; ^3^Faculty of Computational Mathematics and Cybernetics, Moscow State University, Moscow, Russia; ^4^Semenov Institute of Chemical Physics, Russian Academy of Sciences, Moscow, Russia

**Keywords:** transcranial magnetic stimulation, magnetic field, synaptic plasticity, gene expression, magnetoreception

## Abstract

Transcranial magnetic stimulation (TMS) is an effective method used to diagnose and treat many neurological disorders. Although repetitive TMS (rTMS) has been used to treat a variety of serious pathological conditions including stroke, depression, Parkinson’s disease, epilepsy, pain, and migraines, the pathophysiological mechanisms underlying the effects of long-term TMS remain unclear. In the present review, the effects of rTMS on neurotransmitters and synaptic plasticity are described, including the classic interpretations of TMS effects on synaptic plasticity via long-term potentiation and long-term depression. We also discuss the effects of rTMS on the genetic apparatus of neurons, glial cells, and the prevention of neuronal death. The neurotrophic effects of rTMS on dendritic growth and sprouting and neurotrophic factors are described, including change in brain-derived neurotrophic factor concentration under the influence of rTMS. Also, non-classical effects of TMS related to biophysical effects of magnetic fields are described, including the quantum effects, the magnetic spin effects, genetic magnetoreception, the macromolecular effects of TMS, and the electromagnetic theory of consciousness. Finally, we discuss possible interpretations of TMS effects according to dynamical systems theory. Evidence suggests that a rTMS-induced magnetic field should be considered a separate physical factor that can be impactful at the subatomic level and that rTMS is capable of significantly altering the reactivity of molecules (radicals). It is thought that these factors underlie the therapeutic benefits of therapy with TMS. Future research on these mechanisms will be instrumental to the development of more powerful and reliable TMS treatment protocols.

## Introduction

Transcranial magnetic stimulation (TMS) involves the use of alternating magnetic fields to stimulate neurons in the brain and the recording of stimuli-induced responses using electromyography. It has been known for over a century that electricity and magnetism are interdependent. For example, in 1831, Faraday demonstrated that an alternating and rapidly changing magnetic field produces electric currents in an adjacent conductor. In this case, the current runs through a wire coil and generates a magnetic field that is perpendicular to the plane of the coil. In other cases, an electric current can be induced in a conducting medium, such as the brain, when it is exposed to a magnetic field. The preferred direction of the generated current will be parallel but opposite to the current in the primary coil, which is the source of the magnetic field, but the actual currents will strongly depend on the anisotropic and inhomogeneous conductive properties of the medium. Thus, the use of TMS implies electrode-free electric stimulation. In this formulation, the magnetic field acts as an intermediary between the coil and the electric currents that are induced in the brain.

In 1985, this principle was successfully demonstrated in the human cerebral cortex (Barker et al., 1985) and specialized coils that can induce electric currents in any cortical region are currently used for this purpose. The response to single-pulse TMS depends on the cortical region that is stimulated. For example, stimulation of the motor cortex causes contractions in the muscles of the extremities, whereas stimulation of the primary visual cortex induces flashes of light when the eyes of the subject are closed. Over the past 10–20 years, a TMS method known as repetitive TMS (rTMS) has become widely used in clinical neurology. There are two primary rTMS treatment regimes: low-frequency rTMS, which is defined by stimulation at frequencies lower than 1 Hz, and high-frequency rTMS, which is defined by stimulation at frequencies higher than 5 Hz. Low-frequency rTMS reduces neuronal excitability, whereas high-frequency rTMS increases cortical excitability (Maeda et al., 2002).

Large, randomized placebo-controlled studies have demonstrated that rTMS efficaciously treats a variety of pathological conditions and diseases such as depression, obsessive–compulsive disorders, pain syndromes, migraines, refractory epilepsy, tinnitus, Parkinson’s disease, dystonia, tremors, and spasticity. Additionally, rTMS is an effective neurorehabilitation method for patients with sequelae of various nervous system disorders such as central nervous system (CNS) trauma or stroke [for reviews, see Matsumoto and Ugawa (2010), Chervyakov et al. (2012), and Lefaucheur et al. (2014)]. Furthermore, these studies demonstrated that the positive effects of TMS can persist for as long as 6 months after the cessation of treatment. However, although numerous studies have investigated the effects of TMS, two primary issues remain unclear. First, the underlying mechanisms that support the therapeutic effects of rTMS for such a broad range of neuropsychological diseases and conditions have yet to be fully characterized. Second, the reasons for the long duration of the therapeutic effects of TMS following its cessation have yet to be fully elucidated. It is possible that these long-lasting positive effects are related to the actions of TMS at the neural, neural network (mutual excitation and inhibition of cerebral regions), synaptic, and/or molecular genetic (changes in gene expression, enzyme activity, and neuromediator production) levels. Thus, the primary goals of the present review were to evaluate the major effects of rTMS on cellular and molecular genetic processes and to discuss the possible underlying mechanisms supporting these effects.

Several of the initial studies that investigated the effects of electromagnetic fields on biological organisms were conducted in the late nineteenth century in St. Petersburg (Zhadin, 2001). Recently, a vast amount of data regarding the effects of magnetic fields on biological organisms has been accumulated (Levin, 2003; Okano and Ohkubo, 2003; McKay et al., 2007; Pazur et al., 2007; Yamaguchi-Sekino et al., 2011). For example, Pazur et al. (2007) have provided extensive but controversial data concerning the effects of magnetic fields on the frequency of DNA damage, changes in the number of mutations, and enzyme activity using different TMS parameters (frequency, intensity, etc.). Additionally, Kholodov (1982), a professor at the Institute of Higher Nervous Activity in the Russian Academy of Sciences, studied the effects of magnetic fields on the human brain at the Institute of Neurology in the USSR Academy of Sciences. The present review deliberately focuses on the particular effects of TMS-induced electromagnetic fields on neurological processes that occur at different levels in the brain because these effects determine the various therapeutic benefits of this method.

## Effects of rTMS on Neurotransmitters and Synaptic Plasticity

Because the clinical potential of rTMS to treat Parkinson’s disease is very high, a number of experimental studies have investigated the effects of magnetic stimulation on dopamine production. A functional neuroimaging study of patients with Parkinson’s disease revealed that rTMS increased the concentration of endogenous dopamine in the ipsilateral striatum (Strafella et al., 2001). High-frequency (10 Hz) rTMS of the left dorsolateral prefrontal cortex (DLPFC) increases ipsilateral dopamine release in Brodmann areas 25/12 and 32 as well as in Brodmann area 11, which is in the medial orbitofrontal cortex (Cho and Strafella, 2009). These changes in dopamine production resulted in the reduced binding potential of the ligand [11C]FLB 457 during positron emission tomography (PET) scans. However, no significant changes were observed during stimulation of the right DLPFC.

Other studies have produced similar data regarding TMS-induced changes in dopamine production. Theta-burst (high-frequency) stimulation applied to the left DLPFC in healthy volunteers worsened motor performance and reduced bilateral striatal dopamine production (Ko et al., 2008); the ipsilateral caudate nucleus and ipsilateral putamen exhibited the most significant reductions of dopaminergic activity. The effects of this stimulation regime are thought to be associated with its long-lasting inhibition (up to 60 min) of lower-lying brain segments via neuroplastic changes in synaptic structure that likely occur via the activation of NMDA receptors (Huang et al., 2007).

Repetitive TMS also affects the expression levels of various receptors and other neuromediators. Following exposure to rTMS, there is a reduction in the number of β-adrenoreceptors in the frontal and cingulate cortices but an increase in the number of NMDA receptors in the ventromedial thalamus, amygdala, and parietal cortex (Lisanby and Belmaker, 2000). Rats exposed to 5 days of treatment with electromagnetic radiation (60 Hz frequency, 20 G amplitude) exhibit high levels of nitric oxide (NO) and cyclic guanosine monophosphate (cGMP) in the cerebral cortex, gyri, and hippocampus. However, the number and morphology of the neurons remain unchanged. Based on such evidence, it has been proposed that enhanced expression of the genes responsible for synthesis of neuronal NO synthase might underlie the effects of TMS (Cho et al., 2012).

According to current theory, the effects of rTMS are primarily determined by the specific combinations of stimulation frequency and intensity that are used. In response to rTMS, neuronal excitability is altered due to a shift in ionic balance around the population of stimulated neurons (Kuwabara et al., 2002); this shift manifests as altered synaptic plasticity. Most researchers believe that the long-lasting therapeutic effects of rTMS and the effects of magnetic stimulation on the processes described above are related to two phenomena: long-term potentiation (LTP) and long-term depression (LTD; Hoogendam et al., 2010). These processes were first described in the rodent hippocampus.

Long-term potentiation and LTD are thought to be key mechanisms supporting the long-term changes in synaptic strength following exposure to TMS. LTP enhances synaptic strength and can persist for several days, weeks, or months, whereas LTD results in the long-term reduction of synaptic strength (Duffau, 2006; Purves, 2008). LTP is induced by high-frequency, or theta-burst, stimulation or a situation in which the stimulation of a presynaptic neuron is followed by the stimulation of a postsynaptic neuron within several tens of milliseconds. On the other hand, LTD is caused by low-frequency stimulation or the stimulation of a postsynaptic neuron that is followed by the stimulation of a presynaptic neuron within several tens of milliseconds. These changes are not observed when the time difference between the stimulation of the pre- and postsynaptic neurons, in either direction, is longer than 100 ms (Bi and Poo, 1998).

The molecular mechanisms associated with TMS-induced changes likely involve NMDA receptors located on the postsynaptic membrane. NMDA receptors contain a cationic channel that is blocked by magnesium ions during the resting state (Cooke and Bliss, 2006), but a depolarization of the cell membrane eliminates this channel block and allows calcium ions to enter the postsynaptic neuron (Cooke and Bliss, 2006); this eventually leads to the induction of LTP. There are two varieties of the LTP phenomenon: early and late. Early LTP involves a change in synaptic strength following the redistribution of mediator and ion activity and lasts for 30–60 min (Pfeiffer and Huber, 2006). On the other hand, late LTP is associated with altered gene expressions and protein synthesis and can last for several hours, days, or even weeks (Sutton and Schuman, 2006). The activation of NMDA receptors is also involved in LTD but in a different manner. Whereas a rapid postsynaptic increase in calcium ion content induces LTP, the small and slow flow of calcium ions induces LTD (Purves, 2008). For example, magnetic stimulation at 1 Hz reduces an induced muscle response (Wassermann, 1996; Chen et al., 1997; Touge et al., 2001; Maeda et al., 2002; Muellbacher et al., 2002). Additionally, a large-scale review determined that a 15-min rTMS session at 0.9 Hz (800 pulses) with a stimulation intensity of 115% of the motor threshold causes a 20% decrease in the induced muscle response during the subsequent 15-min period (Chen et al., 1997).

Long-lasting low-frequency (1 Hz) stimulation causes pronounced depression that persists for a short period of time, which is consistent with the findings of rodent studies investigating the LTD phenomenon. By contrast, high-frequency stimulation of the primary motor cortex (M1) has been shown to increase cortical activity. In their pioneering study, Pascual-Leone et al. (1994) demonstrated that 20 TMS pulses at a frequency of 20 Hz and an intensity of 150% caused a 50% increase in the induced muscle response within 5 min. Combinations of TMS treatment and pharmacotherapy have also yielded interesting findings. For example, small doses of memantine, a non-competitive NMDA receptor antagonist, may block the relieving effect during LTP (Huang et al., 2007). Similar data have been obtained using d-cycloserine (Teo et al., 2007).

The aforementioned theory is currently considered to be the key working theory of TMS effects. Accordingly, researchers tend to use this theory to interpret virtually any effect of rTMS, including changes in gene expression and neuromediator production. However, other authors have reported that this approach has a number of serious drawbacks (Mally, 2009). Thus, alternative explanations of the therapeutic effects of TMS are discussed in the next section.

## Effects of rTMS on the Genetic Apparatus of Neurons

Ji et al. (1998) demonstrated that a single session of rTMS increased the mRNA expression of c-fos in the paraventricular nuclei of the thalamus and, to a lesser extent, in the frontal and cingulate gyri but not in the parietal cortex. Additionally, this study found that magnetic stimulation had a stronger effect than electric stimulation. By contrast, a 14-day series of rTMS sessions increased the mRNA expression of c-fos in the parietal cortex (Hausmann et al., 2000).

Aydin-Abidin et al. (2008) studied the effects of low- and high-frequency TMS on the genetic expressions of c-Fos and zif268. Low- and high-frequency stimulation enhanced the expression of the c-Fos gene in all tested cortical zones, whereas theta-burst stimulation had similar effects but only in the limbic areas. Theta-burst stimulation also enhanced zif268 expression in all cortical zones, but stimulation at 10 Hz produced this effect in only the motor and sensory cortices. Although stimulation at 1 Hz and sham stimulation did not influence zif268 expression, it is interesting to note that sham stimulation increased c-Fos expression in the limbic zone. Additionally, Funamizu et al. (2005) demonstrated that rTMS influences the expressions of tyrosine hydroxylase and NeuN in the substantia nigra.

An important issue to consider when conducting therapeutic rTMS concerns the types of patient that will be responsive or non-responsive to therapy. Several studies have found that polymorphisms within the genes that encode serotonin (5-HT) carriers, 5-HT_1A_ receptors (Zanardi et al., 2007), and brain-derived neurotrophic factor (BDNF) (Cheeran et al., 2008) influence a patient’s response to therapy. An investigation of the polymorphism within the 5-HT_1A_ receptor gene (*n* = 99; Zanardi et al., 2007) found that C/C patients are more susceptible to TMS therapy than C/G and G/G patients. Another clear illustration of the dependence between genetic polymorphisms and the benefits of TMS is the difference between subjects with the Val66Met and Val66Val alleles of the BDNF gene (Cheeran et al., 2008). Fedi et al. (2008) also studied the effects of mutations in the GABA_A_ receptor gene on cortical susceptibility to TMS signals.

Numerous studies have unambiguously demonstrated that TMS signals stimulate and induce gene expression and enhance the production of a number of enzymes. These effects likely underlie the long-lasting duration of the therapeutic effects of TMS. The effects of rTMS are often stronger than are those of direct electric stimulation, and some changes are only observed following rTMS (Simis et al., 2013).

## Effects of rTMS on Glial Cells and the Prevention of Neuronal Death

Another important aspect of TMS action is its impact on neuroprotective mechanisms. May et al. (2007) morphometrically demonstrated that 1 Hz rTMS applied to the left superior temporal gyrus (Brodmann areas 41 and 42) for 5 days at an intensity of 110% of the TMS motor threshold significantly increased gray matter volume at the stimulation site. No changes in gray matter volume were recorded in patients exposed to sham TMS. These authors suggested that these macroscopic changes were likely dependent on synaptogenesis, angiogenesis, gliogenesis, neurogenesis, increases in cell size, and increases in cerebral blood flow (May, 2011).

Ueyama et al. (2011) showed that 25 Hz rTMS for 14 days enhances neurogenesis in the mouse dentate gyrus, and Meng et al. (2009) found that high-intensity alternating magnetic fields (0.1–10 T) have a positive effect on the differentiation and growth of neural stem cells in the neonatal rat *in vitro*. The maximum effects were attained in the 40,000-G (4 T) field. Following the induction of unilateral damage in the substantia nigra using 6-OHDA, mice exposed to a 60-day period of rTMS treatment exhibited the *in situ* differentiation of neurons in the subventricular zone into dopamine-producing neurons (Arias-Carrión et al., 2004). Moreover, the number of new dopamine-producing cells correlated with enhancements in motor activity. Vlachos et al. (2012) studied the effects of high-frequency (10 Hz) stimulation in the cultured mature hippocampal CA1 cells of mice and found that magnetic stimulation induced a remodeling of the dendritic spines. These effects were related to the impact of TMS on NMDA and AMPA receptors.

Several studies using models of transient ischemic attack and prolonged ischemia found that rTMS protects neurons against death and alters blood flow and metabolism in the brain (Fujiki et al., 2003; Ogiue-Ikeda et al., 2005). rTMS also aids in the recovery of neuronal function following cerebral ischemia-reperfusion injury in rats (Feng et al., 2005). To elucidate the mechanisms underlying these effects, Feng et al. (2008) investigated the effects of rTMS on adenosine triphosphate (ATP) content in the corpus striatum and the expression of microtubule-associated protein-2 (MAP-2) using a model of ischemia-reperfusion injury. rTMS significantly increased ATP content in the striatum of the ischemised hemisphere. Different stimulation regimes induced different effects, but both high- and low-intensity (200 and 120%, respectively) high-frequency stimulation (20 Hz) significantly increased ATP content. Additionally, there was a significant increase in MAP-2 expression in the left ischemised hemisphere and, identical to ATP content, the greatest number of MAP-2-positive zones was observed following high-frequency stimulation.

Gao et al. (2010) studied the neuroprotective effects of high-frequency rTMS in a mouse model of transient ischemic attack using PET imaging. Although the infarct zone was significantly smaller in the affected hemispheres of mice exposed to rTMS, their glucose metabolism was higher. Additionally, the number of caspase-3-positive cells was significantly lower in the rTMS group compared with the control group, which indicates that rTMS inhibited apoptosis in the ischemised zone. Yoon et al. (2011) demonstrated the anti-apoptotic effects of TMS in the areas surrounding the infarct zone in mice; these experimental data aided in the design of clinical protocols that use magnetic stimulation during the acute phase of stroke. Ke et al. (2010) applied low-frequency stimulation to mice prior to the administration of a lithium-pilocarpine mixture (the lithium-pilocarpine model for epileptogenesis) and found an increase in the expression of Bcl-2 but a reduction in the expression of Fas in the hippocampus. This TMS-induced anti-epileptic effect was thought to occur via the activation of anti-apoptotic mechanisms. The latter study is of particular interest because the number of clinical studies investigating the effects of TMS on patients with refractory epilepsy is increasing (Sun et al., 2012).

The neuroprotective effects of TMS are also evident in another animal model that employs the neurotoxin 1-methyl-4-phenyl-1,2,3,6-tetrahydropyridine (MPTH). Although the hippocampal CA3 pyramidal neurons of mice that had not been exposed to TMS were affected 48 h after MPTH treatment, the CA3 pyramidal neurons of mice exposed to rTMS were not. The measurement of glial fibrillary acid protein (GFAP) levels in the astrocytes of mice exposed to TMS revealed that these cells were activated following stimulation (Funamizu et al., 2005). Additionally, astrocytes exhibit an enhanced ability to migrate to a CNS lesion following magnetic stimulation in an animal model of spinal cord injury. This may be due to the activation of specific mitotic pathways (MEK1,2/ERK) and the enhanced expressions of several genes (Fang et al., 2010).

## Neurotrophic Effects of rTMS on Dendritic Growth and Sprouting and Neurotrophic Factors

It is important to note that magnetic stimulation does not necessarily always produce a positive result and that these effects largely depend on the stimulation regime. In hippocampal cell cultures, low-intensity stimulation (1.14 T, 1 Hz) results in dendritic sprouting (axon growth) and growth and increases the density of synaptic contacts (Ma et al., 2013). By contrast, high-intensity stimulation (1.55 T, 1 Hz) has devastating effects that result in decreased numbers of dendrites and axons, the presence of neuronal lesions, and a diminished number of synapses. The authors of this study suggested that these results are associated with the BDNF-tyrosine kinase B (TrkB) signaling system (Ma et al., 2013).

A majority of rTMS studies have focused on changes in BDNF function. BDNF has a molecular weight of 27 kDa and was originally derived from the porcine brain as a trophic factor for cells of the dorsal root ganglia (Leibrock et al., 1989). It was later derived from the human brain as well (Barde et al., 1982). BDNF is known to have a broad range of functions that include enhancements of neuronal survival following CNS damage, neurogenesis, the migration and differentiation of neurons, the growth of dendrites and axons, and synapse formation (Baquet et al., 2004). Recent studies have shown that an external magnetic field, which is a consequence of TMS, may affect BDNF content in the serum and cerebrospinal fluid (CSF), but the data obtained from assessments of serum BDNF levels after TMS sessions are controversial. A number of studies have found that rTMS increases serum levels of BDNF (Yukimasa et al., 2006; Zanardini et al., 2006), but other studies have found no effect (Lang et al., 2006; Gedge et al., 2012). Wang et al. (2011) reported that high-frequency stimulation increases serum BDNF levels and the affinity of BDNF for TrkB receptors, whereas low-frequency TMS reduces BDNF levels. Patients with amyotrophic lateral sclerosis (ALS) exhibit reduced serum levels of BDNF after the application of low-frequency rTMS to the motor cortex (Angelucci et al., 2004), but Yukimasa et al. (2006) demonstrated that high-frequency stimulation increases BDNF levels in the blood plasma of patients with depression.

Prolonged exposure to rTMS (5 days with a 2-day break–11 weeks) significantly increases BDNF mRNA levels in the hippocampus and the parietal and pyriform cortices (Muller et al., 2000). These rTMS-induced effects on the production of neurotrophic factors may explain previously obtained data regarding the neuroprotective and neuroplastic benefits of rTMS, such as the enhanced sprouting of hippocampal mossy fibers (Lisanby and Belmaker, 2000). However, according to some theories, the antidepressant effects of TMS can also be attributed to the influence of rTMS on BDNF production itself (Ogiue-Ikeda et al., 2003a,b). The BDNF proteins that are synthesized under the magnetic field induced by TMS exhibit all of the usual desired properties and, thus, BDNF is thought to be protective of synaptic transmission following ischemic brain injury (Ogiue-Ikeda et al., 2005).

Hence, it has been reliably demonstrated that rTMS affects BDNF production in stimulated as well as remote brain regions. These findings offer a variety of novel possibilities regarding therapeutic options for patients with CNS disorders. Table 1 summarizes the main findings of the studies that have investigated the effects of TMS on the CNS. The effects of magnetic stimulation influence a variety of factors including neuronal morphology; glial cells; neurogenesis; cell differentiation and proliferation; apoptotic mechanisms; the concentrations of neuromediators, ATP, and neurotrophic factors; glucose metabolism; and the expression of certain genes. The clinical significance and positive therapeutic effects of rTMS are likely determined by various combinations of these factors. Although there is a significant amount of published data regarding these effects, its precise mechanisms remain unclear. This raises questions regarding whether the rTMS-induced magnetic field exerts specific actions or has non-specific actions that are based on some type of non-electrical mechanisms that have yet to be considered. The next section of this review focuses on these putative mechanisms.

**Table 1:** **Effects of magnetic stimulation**.

Study	Study group	Object of study	Stimulation regime	Effect
**EFFECTS OF rTMS ON NEUROTRANSMITTERS**				
Strafella et al. (2001)	Patients with Parkinson’s disease	Functional neuroimaging (PET) of dopamine level		Increased concentration of endogenous dopamine in the striatum of ipsilateral hemisphere
Cho and Strafella (2009)	Patients with Parkinson’s disease	Binding potential of [11C]FLB 457 ligand during PET	High-frequency (10 Hz) stimulation of the left DLPC	Increased dopamine release in ipsilateral Brodmann areas 25/12; 32 and in the medial orbitofrontal cortex
Ko et al. (2008)	Healthy volunteers	PET	High-frequency theta-burst stimulation (TBS) of the left DLPC	Bilaterally reduced dopamine production in the striatum
Cho et al. (2012)	Rats	Levels of neuromediators NO and cGMP (cyclic guanosine monophosphate)	Electromagnetic radiation for 5 days (60 Hz frequency, 20 G amplitude)	High-neuromediator level was observed in the cerebral cortex, gyri, and hippocampus after stimulation, while the number and morphology of neurons remained intact
Lisanby and Belmaker (2000)	Mice	Expression of cortical β-adrenoreceptors and NMDA receptors		Reduced number of β-adrenoreceptors in the frontal and cingulate cortex is reduced. Increased number of NMDA receptors in the ventromedial thalamus, amygdala, and parietal cortex
**EFFECT OF rTMS ON THE GENETIC APPARATUS OF NEURONS**				
Ji et al. (1998)	Mice	c-fos mRNA	Single rTMS session	Enhanced c-fos mRNA expression in thalamic paraventricular nuclei in the frontal and cingulate gyri
Hausmann et al. (2000)	Mice	c-fos mRNA	14-day series of rTMS sessions	Enhanced c-fos mRNA expression in the parietal cortex
Aydin-Abidin et al. (2008)	Mice	Expression of the c-Fos and zif268 genes	High- and low-frequency stimulation and TBS	Low- and high-frequency stimulation enhanced c-Fos expression in all the tested cortical regions. TBS enhanced c-Fos expression in the limbic region. zif268 expression increased in all the cortical regions after TBS and in the motor and sensory cortex after high-frequency stimulation
Funamizu et al. (2005)	Mice	Expression of tyrosine hydroxylase and NeuN in the substantia nigra		rTMS enhanced expression of tyrosine hydroxylase and NeuN in the substantia nigra
**BIOLOGICAL EFFECTS OF rTMS ON NEUROPROTECTION (PREVENTION OF CELL DEATH) AND GLIAL CELLS**				
May (2011)	Healthy volunteers	Gray matter volume according to morphometric data	1 Hz, 110% of the motor threshold, 5 days	Increased gray matter volume in the stimulated zone
Vlachos et al. (2012)	Mouse hippocampal cell culture (CA1)	Neuronal morphology	10 Hz	Dendritic spine remodeling
Ueyama et al. (2011)	Mice	Neurogenesis in the dentate gyrus	25 Hz	Enhanced neurogenesis in the dentate gyrus
Meng et al. (2009)	Cell culture		High-intensity (0.1–10 T) alternating magnetic field	Positive effect on growth and differentiation of neuronal stem cells
Guo et al. (2014)	Rats	Proliferation of adult neural stem cells (NSCs) and explored microRNAs (miRNAs) after focal cerebral ischemia	10 Hz	10 Hz rTMS significantly increased the proliferation of adult NSCs after focal cerebral ischemia in the subventricular zone (SVZ)
				10 Hz rTMS can promote the proliferation of adult NSCs in the SVZ after focal cerebral ischemia by regulating the miR-25/p57 pathway
Fujiki et al. (2003), Ogiue-Ikeda et al. (2005), and Feng et al. (2008)	Mouse model of transient ischemia	Neuronal morphology, number of neurons		Neuroprotective effect of TMS
Feng et al. (2008)	Mouse model of transient ischemia	ATP level and expression of microtubule-associated protein	20 Hz	Increased ATP level and enhanced expression of microtubule-associated protein in the ipsilateral hemisphere
Gao et al. (2010)	Mouse model of transient ischemia	PET (glucose metabolism), infarct size		Reduced infarct size and increased glucose metabolism in the affected area
Gao et al. (2010)	Mouse model of transient ischemia	The number of caspase-3 positive cells (apoptotic marker)		Significant reduction of the number of caspase-3 cells, which indicates that rTMS affects inhibition of apoptosis in the ischemized zone
Yoon et al. (2011)	Mouse model of transient ischemia	Apoptotic markers		The anti-apoptotic effect of TMS on the zone surrounding an infarct in mice
Ke et al. (2010)	Lithium-pilocarpine-induced mouse model of epileptogenesis	Apoptotic markers	Low-frequency stimulation	Enhanced Bcl-2 expression and reduced Fas expression. Activation of anti-apoptotic mechanisms
Funamizu et al. (2005)	MPTP-induced mouse model of neurodegeneration	The number of CA3 pyramidal neurons		No damage to CA3 pyramidal neurons was observed when rTMS was performed 48 h after neurotoxin administration
Funamizu et al. (2005)	MPTP-induced mouse model of neurodegeneration	Measurement of glial fibrillar acidic protein in astrocytes		rTMS activates astrocytes
Fang et al. (2010)	Mouse models of spinal cord injury	Migratory ability of astrocytes		Magnetic stimulation induced increased ability of astrocytes to migrate toward the CNS damage focus
**THE NEUROTROPHIC EFFECTS OF rTMS (DENDRITIC GROWTH AND SPROUTING, NEUROTROPHIC FACTORS)**				
Ma et al. (2013)	Mouse hippocampal cell culture	Neuronal morphology	(1) Low-intensity (1.14 T, 1 Hz)	Low-intensity stimulation results in sprouting and dendrite growth, increases the density of synaptic contacts
			(2) High-intensity (1.55 T, 1 Hz)	High-intensity stimulation (1.55 T, 1 Hz) reduces the number of dendrites and axons, induces structural lesions in neurons, and reduces the number of synapses
Lisanby and Belmaker (2000)	Mice	Hippocampus		Enhanced sprouting of hippocampal mossy fibers
Yukimasa et al. (2006) and Zanardini et al. (2006)	Healthy volunteers	Serum BDNF concentration		Increased BDNF serum level
Lang et al. (2006) and Gedge et al. (2012)	Healthy volunteers	Serum BDNF concentration		No effect
Wang et al. (2011)	Healthy volunteers	Serum BDNF concentration	(1) Low-frequency	The BDNF level increased after high-frequency stimulation and decreased after low-frequency TMS
			(2) High-frequency	
Angelucci et al. (2004)	Patients with amyotrophic lateral sclerosis	Serum BDNF concentration		Reduced BDNF level in blood plasma
Yukimasa et al. (2006)	Patients with depression	Serum BDNF concentration	High-frequency stimulation	Increased BDNF concentration in blood plasma
Muller et al. (2000)	Mice	BDNF mRNA	rTMS (5 days with a 2-day break – 11 weeks)	Increased level of BDNF mRNA in hippocampus, parietal, and piriform cortex

## Non-Classical Effects of TMS Related to Biophysical Effects of Magnetic Fields

Prior to beginning this section, it is important to mention that the relationships among the theories proposed below and the effects of TMS remain unproven and have yet to be verified. Nevertheless, these phenomena have been shown to underlie the impact of induced magnetic fields on various physical, chemical, and biological systems in various circumstances. It is suggested here that these factors may be significant contributors to the TMS effects described above, including the long-lasting effects.

### Quantum effects

Electromagnetic fields in biological systems act on charged and magnetic particles that are often of microscopic size; thus, it is natural to expect that the action of TMS on the brain may be governed by quantum laws at the most basic levels. Because a number of macroscopic quantum phenomena exist (Leggett, 2002), many physicists currently believe that quantum effects play a crucial role in the macroworld, including in organic (biological) systems (Vedral, 2008). There are several approaches to understanding mind and brain that involve quantum descriptions. Penrose, a well-known English physicist, is among the researchers investigating this subject, and he and Hameroff developed a theory that proposes that quantum measurement plays an important role in consciousness and that microtubules act as carriers of quantum information (Penrose and Hameroff, 2011).

Research into the actions of quantum effects in biological systems has gradually become an individual discipline known as quantum biology. Presently, this discipline is in its embryonic stages, but recent findings indicate that it is a promising field of research that may produce great advances in the future. For example, the thorough examination of photosynthesis in the alga Chroomonas CCMP270 performed by Collini et al. (2010) revealed that the electrons in pigment proteins responsible for the absorption of photons at a certain frequency acquire a state of quantum superposition, in which they remain for a long time (400 fs), when they are in excited states.

The magnetic compass orientation in birds is another illustration of the direct effects of magnetic fields. For example, birds exposed to an external magnetic field are unable to differentiate between north and south (Wiltschko and Wiltschko, 2010). To interpret this phenomenon, Ritz et al. (2010) suggested that magnetoreception in birds is based on a central phenomenon of quantum mechanics known as quantum entanglement, which allows particles to remain interconnected despite spatial separation. There are special molecules in the eyes of birds that have two electrons that form an entangled pair with zero total spin. These electrons are separated after the molecule absorbs a quantum of light but quantum entanglement is retained, and this configuration becomes highly sensitive to external factors, including magnetic fields. Tilted magnetic fields have different effects on electron pairs and can induce an imbalance that alters the chemical reactions in which the molecule can participate. The chemical processes that occur in the bird eye transform these differences into nerve impulses that form an image of the magnetic field in the brain.

It should be mentioned that the lifetimes of the biological quantum effects of both photosynthesis and magnetoreception in birds are longer than are those of quantum mechanics experiments performed in the laboratory under similar conditions. The aforementioned examples and the theories proposing that quantum systems play a crucial role in brain functioning indicate that the effects of TMS-induced magnetic fields should be considered at this level as well. This may be particularly true concerning the possible effects of these fields on electron pairs, excited states, magnetic nuclei, and any macroscopic quantum phenomena in the CNS.

### Magnetic spin effects

The central idea of the proposed theory is that the magnetic field induced during rTMS has particular effects on living cells and that these effects are not directly related to electrical processes. The spin of electrons is a potential target of magnetic fields in the brain, and a rTMS-induced magnetic field can change the state of electronic spin systems (radicals, ions, or triplet molecules), which, in turn, can influence the chemical activity of corresponding compounds. Molecules with non-zero electronic spin play a variety of important roles in biochemical processes including conjugating phosphorylation reactions and participating in enzyme-catalyzed reactions, gene expression, redox reactions with iron and copper ions, and electron transport along the cytochrome chain (Buchachenko, 1980). Experimental evidence suggests that magnetic fields influence these processes, particularly the effects of a magnetic field on the synthesis of ATP molecules (Buchachenko and Kuznetsov, 2006).

It is important to discuss the molecular mechanisms related to spin dynamics in greater detail. In addition to weight and charge, elementary particles, such as electrons and protons, are characterized by an intrinsic angular momentum called spin, and they therefore have an associated spin magnetic momentum. The magnetic properties of atoms are determined primarily by the spin of electrons, and the spins in chemical reactions are strictly conserved. This fundamental law implies that chemical reactions are spin-selective (i.e., they allow for only those spin states of reagents that coincide with the spin of the products, and they are forbidden if the spin needs to be changed) (Buchachenko, 2009). This law has been experimentally proven (Step et al., 1992).

**Figure 1:**
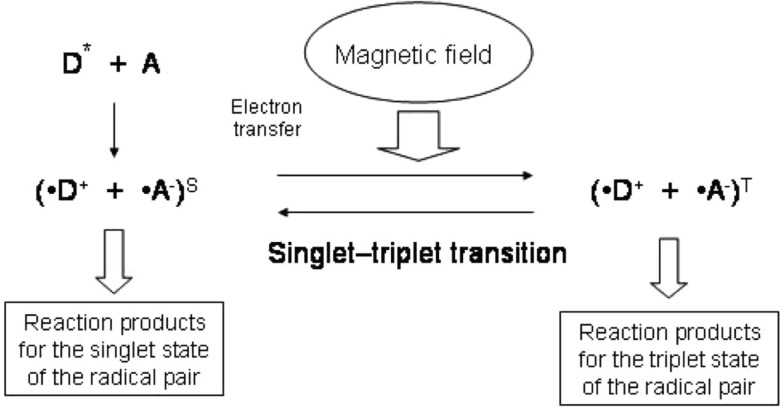
**Scheme of the reaction involving radical pairs with yield of the reaction products depending on magnetic field**. Electron transfer from a donor molecule (D) to an acceptor molecule (A) gives rise to a radical pair. External magnetic field affects the transition between the singlet and triplet states of the radical pair.

A radical pair may have two spin states: the singlet, which has zero total spin, or the triplet, in which the spins of two electrons are added to yield one spin. The reaction that yields the diamagnetic molecule R–R with zero spin is possible only for the singlet spin state, and this mechanism is spin allowed, whereas the triplet mechanism is forbidden (Figure 1; Buchachenko, 2009). According to the definition proposed by Buchachenko (2014), a pair of reagents in these reactions acts as a spin-selective nanoreactor and, by controlling the spin of the nanoreactor, one can switch the reaction from the spin forbidden to the spin-allowed channel. Spin-selective reactions are also sensitive to magnetic fields such that spin states can be changed only by magnetic interactions. Magnetic fields can induce triplet–singlet spin transitions in these pairs and change their total spin state and reactivity. This concept underlies spin chemistry and spin biology (Salikhov et al., 1984; Buchachenko and Frankevich, 1994; Buchachenko, 2009, 2014). It has been hypothesized that these effects give rise to all the major magnetic effects in both chemistry and biology (Buchachenko, 2014). More specifically, this would mean that the proper magnetic effects of TMS on the brain are mediated by its influence on the spin chemistry of biochemical reactions.

Recent studies have revealed that the ion-radical mechanisms involved in two enzyme-catalyzed reactions that are of fundamental significance: ATP synthesis and DNA synthesis, including replication and effects on DNA polymerase (Buchachenko et al., 2005, 2008; Buchachenko and Kuznetsov, 2006). Both types of reactions ensure the normal functioning of living organisms and are catalyzed by complexes that include metal ions such as magnesium and zinc. Major differences in the catalytic activity of ions with magnetic nuclei (e.g., ^199^Hg, ^25^Mg, ^67^Zn, and ^43^Ca) have been found in both types of reactions. This phenomenon, called the magnetic isotope effect, is indicative of the ion-radical reaction mechanism described above that can be controlled by magnetic fields and, thus, may be sensitive to TMS. In fact, a number of studies have reported that the effects of rTMS on the genetic apparatus of a cell may be the result of TMS-induced magnetic fields on DNA polymerase via the mechanisms described above. Accordingly, Feng et al. (2008) demonstrated the effects of TMS on ATP levels, which can also be directly explained by the effects of a TMS-induced magnetic field on spin systems.

The effects of a stimulation regime on this mechanism are important to consider. In a spin nanoreactor, rTMS frequencies ranging from 1 to 30 Hz have the same effect as a constant field and cause only spin dephasing, which leads to singlet–triplet conversion (Buchachenko, 2014). This effect is associated with the positive impacts of TMS under different stimulation regimes. On the other hand, the differential effects of high- and low-frequency rTMS have been demonstrated by a number of studies. According to the proposed theory, these differences should be attributed to the electrical effects of TMS.

Nuclear spin also plays a significant role in chemical reactions. The rate of a chemical reaction depends on the magnetic momentum of reagent nuclei that controls the reactivity via magnetic interactions between the electron and the nucleus (Buchachenko, 2014). The magnetic nucleus generates a constant local magnetic field around the electron that makes the electronic spin process with an additional velocity. As mentioned above, magnetic interactions are unique and only these types of interactions can change the state of a reagent’s spin system in a chemical reaction. Because magnetic interactions are characterized by negligibly low energy, they eliminate spin-forbiddenness by making the reactions in radical pairs with the spin-forbidden mechanism spin-allowed. This mechanism can explain the influence of magnetic fields on biochemical reactions, which may be one of the mechanisms supporting the effects of TMS on the brain.

### Genetic magnetoreception

The effects of magnetic fields on the genome have been recognized for a significant period of time but it is extremely important to identify the acceptor of this type of field in the genetic apparatus to confirm and study the genome-regulating effects of a magnetic field. According to one theory, a weak magnetic field can induce certain genetic effects due to the actions of proteins belonging to the cryptochrome (CRY)/photolyase family, which are magnetosensitive inhibitors of transcription factors. Experiments on the *Arabidopsis thaliana* plant have demonstrated the involvement of CRYs in biological reactions following changes in a magnetic field (Lin and Todo, 2005).

Cryptochromes are ancient regulatory proteins that are susceptible to electromagnetic radiation and constant magnetic fields. These proteins are classified as flavoproteins, have molecular weights ranging from 50 to 70 kDa, and are present in virtually all living organisms, including the cells of bacteria, plants, insects, and animals (Ahmad et al., 2007). CRYs are expressed in most organs and tissues in these organisms, but they are predominantly localized in the cell nucleus (Lin and Todo, 2005). In 2000, it was hypothesized that CRYs contain magnetosensitive radical pairs (Ritz et al., 2000). Furthermore, it is believed that the activation or inhibition of functional CRY activity is caused by conformational changes at the active site of the protein, which, in turn, influence its interaction with the next element in the signaling pathway (Lin and Todo, 2005; Partch and Sancar, 2005). These authors concluded that CRYs are unique components of living systems that combine biosensory and bioregulatory functions, act as intermediates between living nature and the physical environment, and provide living organisms with the abilities to respond to magnetic and electromagnetic fields and adjust their biological clocks to diurnal and other physical variations in the environment. It is possible that the mechanisms supporting magnetoreception were somehow conserved in humans during evolution or that they have evolved into another mechanism that is indirectly influenced by TMS-induced magnetic fields.

### Macromolecular effects of TMS

Macromolecular mechanisms are typical of processes involving large molecules or organelles. These elements can be oriented or deformed under the influence of a magnetic field, and this type of change alters their properties and reactivity (Buchachenko, 1980). Based on this theory, Bingi and Savin (2003) proposed that the primary interactions between a magnetic field and various particles, electrons, atoms, and molecules are purely physical processes. The charged particles in living matter, such as the ions and molecules that are involved in biophysical and biochemical processes, seem to act as intermediates to the next biological level during the transduction of magnetic signals. Additionally, the fine adjustments in protein activity that are performed by biophysical mechanisms involve ions and intermediary molecules that shift metabolic processes (Bingi and Savin, 2003). Data regarding these effects on voltage-gated ion channels clearly illustrate the macromolecular effects of magnetic fields (Pall Martin, 2013; Lu et al., 2014).

It has been hypothesized that low-intensity magnetic fields disturb ionic conductivity through the membranes of excitable tissues, but it has also been suggested that ion channels contain ferroelectric transmembrane inclusions in the form of either crystals or liquid crystals. Proteins in the plasma membrane are capable of rectifying oscillating electric fields and the constant electrical current that is generated transports compounds through the membrane; this ability of membranes to average signals is essential for an accurate understanding of the effects of magnetic fields on cellular systems. These interactions generate electrical forces that are similar in strength to those induced by normal voltages across the membrane due to the action of ion channels. Hence, magnetic fields regulate currents via cationic channels by changing steady-state cation concentrations in cells; thus, they can influence metabolic processes depending on the concentration of cations (Pall Martin, 2013; Lu et al., 2014).

Although a complete characterization of the effects of magnetic fields has yet to be achieved and the underlying mechanisms are not yet fully understood, the effects of TMS cannot be ignored.

### Electromagnetic theory of consciousness

The electromagnetic theory of consciousness originally proposed by McFadden (2002) suggests that an electromagnetic field induced by the brain is the carrier of consciousness and conscious experience. The starting point of this theory is that every time a neuron generates an action potential it also generates a disturbance in the surrounding electromagnetic field. Thus, it appears that the information encoded by the firing patterns exhibited by excited neurons affects the electromagnetic field of the brain (McFadden, 2002). Further studies are necessary to either confirm or refute this hypothesis. However, it is interesting to note that some researchers try to use rTMS in patients with brain injuries and altered states of consciousness, such as a persistent vegetative state or a minimally conscious state (Louise-Bender Pape et al., 2009; Manganotti et al., 2013; Guerra et al., 2014). There are both positive and negative results reported in these articles. Hence, according to the guidelines on the therapeutic use of rTMS (2014), there is no evidence for a therapeutic effect of rTMS in vegetative states, at least with conventional coils and current safety parameters (Lefaucheur et al., 2014).

## Possible Interpretations of TMS Effects According to Dynamical Systems Theory

The previous sections of the present review have focused primarily on the local physical effects of TMS, but this type of stimulation also influences long-distance functional connectivities (Eldaief Mark et al., 2011) and generates contralateral responses (Komssi et al., 2002). These findings indicate that the brain is affected by TMS as a global dynamical system and, thus, it is necessary to evaluate the effects of TMS in terms of modern dynamical theory. Accordingly, this section discusses the mechanisms that are peculiar to complex non-linear systems and that may be responsible for the therapeutic effects of TMS. Unfortunately, due to the fundamental unpredictability property of non-linear systems, dynamical systems theory does not lead to exact calculations or precise modeling. However, this theory postulates that all such systems are characterized by common principles and typical scenarios during the course of their dynamics, and this allows for qualitative analyses of a problem. Thus, two typical properties of dynamic systems will be taken into account in the present discussion: different time scales and attractors.

The set of parameters that describe a system can often be subdivided into different groups according to their change rate. In the CNS, fast parameters are associated with states of neuronal electrical activity, and slow parameters are associated with the morphological and molecular states of neurons. At small time values, slow parameters are considered to be constants. The trend toward an attracting set of states, or an attractor, is also a typical behavior of dynamical systems. A system may have several attractors with respective domains (basins) of attraction; this means that if the state of a system is in the basin, the system reaches the correspondent attractor after a period of time. There are different types of attractors, including a stationary point (a certain fixed state), a limit cycle (the oscillatory regime), a limit torus (conditionally periodic motion with several frequencies), and the so-called strange attractor, which has a fractal structure (Lorenz, 1963). In this context, a key assumption is that the current pathology of a patient can be explained by the fact that his or her nervous system remains in this attractor state. Thus, it is possible to refer to a *pathological attractor* in neuronal activity dynamics, which is a concept that is very similar to the term *stable pathological condition* (Bekhtereva, 1979, 1999; Vasilevskii, 1986; Fröhlich et al., 2010).

**Figure 2:**
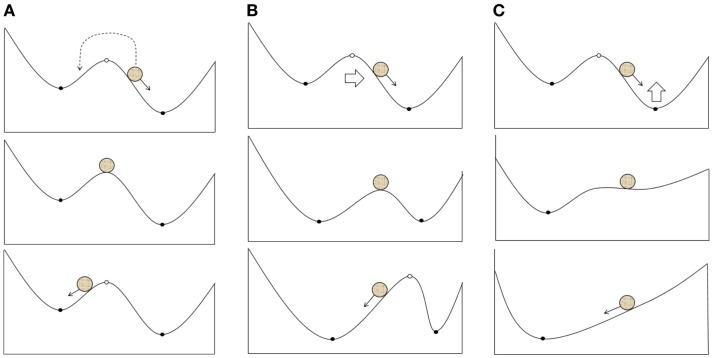
**Possible scenarios of the system leaving the pathological attractor state due to a therapeutic external impact: (A)** transition of the current state from the pathological to the normal attractor basin; **(B)** expansion of the normal attractor basin to include the current state; **(C)** bifurcation resulting into the extinction of the pathological attractor.

Based on the pathological attractor hypothesis, TMS therapy may include external impacts on the slow or fast variables of a system that can move them away from an attractor. This process may follow different scenarios:

1. The transition of a system between basins of different attractors due to the effects on fast variables (electrical activity; Figure 2A). In particular, it is reasonable to suggest this scenario when using single TMS pulses or a short series of pulses.
2. A shift in the attractors of a system due to the effects on slow variables, which result in the current state falling into a new basin of attraction (Figure 2B); however, the overall structure of attractors is retained. LTP may be one of the mechanisms underlying these changes.
3. Changes in the structure of attractors in a system (bifurcation) due to the impact of slow variables on the current attractor (Figure 2C). The structure of the possible regimes of neuronal activity is changed in this case, and these changes can be induced by either LTP or changes in neuronal morphology and gene expression.

The properties of these scenarios are similar to those of the dynamics observed when neurological disorders are treated by stimulation therapy. Thus, Bekhtereva (1999) described the brain processes induced by electrical stimulation therapy as follows:

1. “When I was analyzing these observations, I had a feeling as if we were rocking some type of a barrier and eventually broke it or overcame it” and
2. “… some physiological parameters of the brain state confirm that it has switched to a new functioning mode” (Bekhtereva, 1999).

In the terminology of the present review, “breaking” the barrier corresponds to Scenario 3 (bifurcation), whereas “overcoming” the barrier corresponds to either Scenario 1 (the current state crosses the barrier) or Scenario 2 (the barrier is shifted with respect to the current state).

The relationships among the typical scenarios of dynamical systems behavior and the effects of neurological therapy demonstrate the importance of studying the dynamical properties of TMS. For example, these relationships may explain the extended duration of positive TMS effects because there is a strong possibility that only the basin of the pathological attractor will be reached in Scenario 1. Subsequently, this would lead to the re-emergence of clinical symptoms after some time. The dynamical systems approach may also aid in determining an appropriate treatment protocol because if the inevitability of Scenarios 2 or 3 were known, protocols capable of inducing plastic changes would be used. If Scenario 1 is sufficient, short impacts that make the system relocate between the basins of attraction can be used. Furthermore, the consideration of negative scenarios, such as increasing the basin of a pathological attractor, may reduce the risk of aggravating a patient’s condition. However, despite its positive outlook, it is important to note that the usage of dynamical systems theory to understand the effects of TMS requires serious additional theoretical and experimental work.

## Conclusion

**Figure 3:**
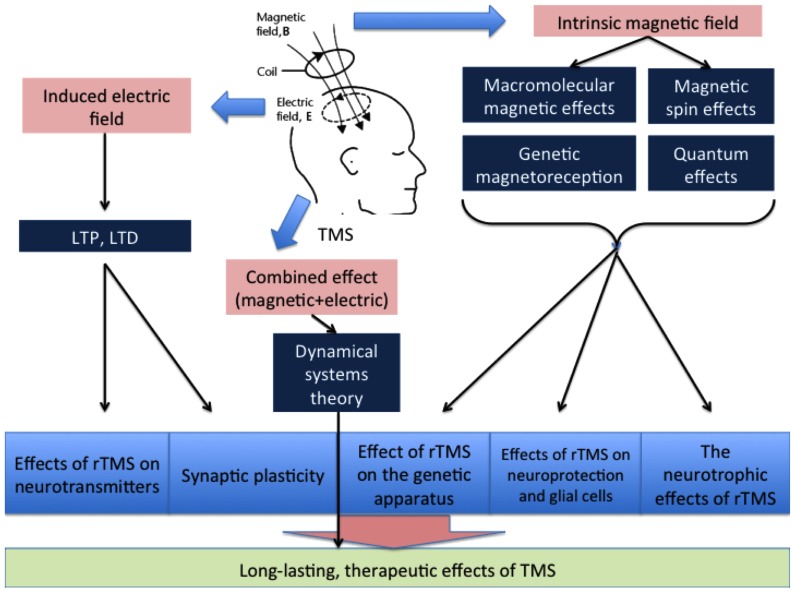
**The general scheme of the influence of magnetic and electric fields**.

The total therapeutic effects of rTMS may be determined by their total impact on a number of processes in the brain, including LTP, LTD, changes in cerebral blood flow, the activity of certain enzymes, interactions between cortical and subcortical structures, and gene expression. A simplified diagram of the effects of TMS on these processes is provided in Figure 3. The location of the impact, the intensity and frequency of the stimulation, and the protein and physicochemical conditions of the stimulated area are also of particular significance when determining the effects of TMS therapy.

The magnetic field induced by rTMS should be regarded not only as a painless transmission of electrical signals to neurons but also as a separate physical factor that has an influence at the subatomic level. This gives rTMS the ability to significantly change the reactivity of molecules (radicals) and produce therapeutic effects. Future studies should explore these effects in greater detail.

## Conflict of Interest Statement

The authors declare that the research was conducted in the absence of any commercial or financial relationships that could be construed as a potential conflict of interest.
